# Child and Adolescent Crisis Care Program: Clinical Profile and Outcomes After Intervention

**DOI:** 10.1192/j.eurpsy.2026.11163

**Published:** 2026-07-31

**Authors:** C. Diaz Tellez, M. Artigas Dorado, I. Quiñoa Jimenez, R. M. Calvo Escalona

**Affiliations:** Hospital Clinic, Barcelona, Spain

## Abstract

**Introduction:**

In recent years, there has been a shift toward community-based approaches to manage psychopathological crises in children and adolescents at home. This poster presents the implementation of the *Child and Adolescent Crisis Care Program* at Hospital Clínic de Barcelona, aimed at providing intensive home and community care. Eligible patients include those with suspected or confirmed mental disorders and/or acute psychopathological crises, psychosocial risk, or social complexity that limits access to usual care or requires intensified support.

**Objectives:**

To analyze the clinical impact of the home-based intensive care program and describe the profile and complexity of patients requiring these services.

**Methods:**

A retrospective study of patients treated and discharged during the program’s first two years (November 2022–November 2024).

**Results:**

Fifty-one cases were attended. Patients showed high scores for emotional neglect and abuse (CTQ mean scores: emotional abuse 11.6 ± 5.68; emotional neglect 11.58 ± 4.44). Post-intervention comparisons revealed significant reductions in overall distress (p=0.032) and suicidal ideation (p=0.022), improvement in global functioning (self-report p=0.28; therapist-rated p<0.001), and enhanced family relationships (self-report, parents, and therapists p<0.001). School absenteeism decreased significantly (self-report p=0.024; parents p=0.007; therapists p<0.001). Satisfaction was high among patients (mean 4.57/5) and families (mean 4.85/5), with strong ratings for perceived usefulness and improvement.

**Conclusions:**

This program proves effective for highly complex cases, improving global functioning, symptomatology, family relationships, and school attendance. Its multidisciplinary, intensive, and home-based approach provides a comprehensive view of patient needs and offers a valuable alternative to inpatient care.

**Disclosure of Interest:**

None Declared

